# Physiological and Transcriptomic Dynamics in Mulberry: Insights into Species-Specific Responses to Midday Depression

**DOI:** 10.3390/genes15121571

**Published:** 2024-12-05

**Authors:** Yong Li, Jin Huang, Fangyuan Song, Zhiyue Guo, Wen Deng

**Affiliations:** Cash Crops Research Institute, Hubei Academy of Agricultural Sciences, Wuhan 430064, China; liyong8057@hbaas.com (Y.L.); huang_jin@hbaas.com (J.H.); 18754861147@163.com (F.S.); gzy15871035162@163.com (Z.G.)

**Keywords:** mulberry, midday depression, RNA-seq, physiological response, photosynthesis

## Abstract

**Background/Objective:** The midday depression of photosynthesis, a physiological phenomenon driven by environmental stress, impacts plant productivity. This study aims to elucidate the molecular and physiological responses underlying midday depression in two mulberry species, Ewu No. 1 (Ew1) and Husan No. 32 (H32), to better understand their species-specific stress adaptation mechanisms. **Methods:** RNA-seq analysis was conducted on leaf samples collected at three time points (10:00 a.m., 12:00 p.m., and 4:00 p.m.), identifying 22,630 differentially expressed genes (DEGs). A comparative Kyoto Encyclopedia of Genes and Genomes (KEGG) pathway analysis was performed to reveal the involvement of key metabolic and signaling pathways in stress responses. **Results:** Ew1 displayed enhanced stress tolerance by upregulating genes involved in energy management, water conservation, and photosynthetic processes, maintaining higher photosynthetic rates under midday stress. In contrast, H32 adopted a more conservative response, downregulating genes related to photosynthesis and metabolism, favoring survival at the expense of productivity. The KEGG analysis highlighted starch and sucrose metabolism and plant hormone signaling as critical pathways contributing to these species-specific responses. **Conclusions:** Ew1’s adaptive molecular strategies make it more suitable for environments with variable light and temperature conditions, while H32’s conservative approach may limit its productivity. These findings provide valuable insights for breeding programs aimed at improving stress tolerance and photosynthetic efficiency in mulberry and other crops, particularly under fluctuating environmental conditions.

## 1. Introduction

Photosynthesis is a critical process that underpins crop yield and the quality of a plant’s dry matter production [1]. The efficiency of photosynthesis is directly linked to crop productivity, with a well-established positive correlation between leaf photosynthetic rate and overall yield [2,3]. In many plant species, the diurnal variation in net photosynthetic rate typically follows a bimodal pattern, characterized by peaks in the morning and afternoon and a notable decline during midday—a phenomenon known as the midday depression of photosynthesis [4,5]. This midday depression is primarily attributed to a combination of genetic traits and environmental factors, including intense light, elevated temperatures, low humidity, and soil drought, which induce partial stomatal closure and increase photorespiration or photoinhibition, ultimately reducing photosynthetic efficiency [6,7,8].

Under natural conditions, the diurnal variation in plant photosynthesis generally adheres to one of two distinct patterns: a unimodal curve, where the highest photosynthetic rate occurs at midday, or a bimodal curve, marked by peaks in the morning and afternoon with a noticeable decline at midday, referred to as midday depression [5,7]. This phenomenon is widespread, occurring across various plant types, including C3, C4, and CAM plants [9]. Despite its prevalence, the precise mechanisms driving midday depression remain poorly understood, making it a focal point of recent research in the field of photosynthesis. Understanding these mechanisms is crucial for developing strategies to enhance plant productivity, particularly in the context of global climate change and the need for more efficient agricultural practices.

The midday depression of photosynthesis is a well-documented physiological phenomenon that significantly impacts plant productivity, particularly in C3 crops. This decline in photosynthetic rate during midday is influenced by a complex interplay of ecological, physiological, and biochemical factors [10,11]. Ecological factors such as light intensity, temperature, relative humidity, and CO_2_ concentration act as external triggers, leading to partial stomatal closure and reducing net photosynthetic rates [12,13]. Physiological factors, including stomatal conductance, mesophyll resistance, leaf water potential, vapor pressure deficit, and physiological rhythms, further modulate this response [14]. Additionally, biochemical factors such as the accumulation of photosynthetic products, RuBP carboxylase activity, and photochemical system activity contribute to the overall decline in photosynthesis during midday [15].

Photoinhibition, a key aspect of midday depression, results from the absorption of excess light energy by the photosynthetic apparatus, leading to a reduction in photosynthetic function [16]. The severity of photoinhibition correlates with the intensity of excess light, and it is estimated that even in the absence of other environmental stressors, photoinhibition can decrease photosynthetic productivity [17]. Photorespiration plays a critical role in managing excess light energy, although it does so at the expense of organic matter, reducing photosynthetic efficiency in C3 crops [18]. While midday depression has evolved as an adaptive mechanism to help plants survive under adverse environmental conditions, it leads to the suboptimal utilization of solar energy during the period when sunlight is most abundant [19,20,21].

Mulberry (*Morus alba* L.), a species originating from China, has gained significant attention in recent years due to its versatile applications in food, medicine, animal feed, and ecological restoration, largely attributable to its rich nutritional profile and functional properties [22,23]. As a typical C3 plant, mulberry’s diurnal photosynthetic activity typically follows a bimodal pattern, characterized by two peaks—in the morning and afternoon—interspersed by a midday depression [24]. However, recent evaluations of 116 mulberry germplasm resources from Shennongjia and other regions in Hubei Province, preserved at the Central China Sub-center of the National Mulberry Germplasm Repository, have identified a rare deviation from this pattern. Specifically, three female plants (Ewu No. 1, Ewu No. 2, and Zhushan No. 2) and one male plant (Ejian 82-12) were found to exhibit a unique unimodal curve of net photosynthetic rate, lacking the typical midday depression. The molecular mechanisms responsible for this unusual photosynthetic behavior remain largely unexplored. Understanding the factors that enable these particular mulberry varieties to maintain consistent photosynthetic rates throughout the day, without the midday decline, could provide valuable insights into improving photosynthetic efficiency in other crop species. This study seeks to investigate the underlying molecular and physiological mechanisms driving this phenomenon, with the potential to inform future breeding programs aimed at enhancing crop resilience and productivity. By uncovering these mechanisms, it may be possible to develop new strategies to optimize photosynthetic performance in mulberry and other economically important crops, thereby contributing to sustainable agricultural practices in a changing global environment.

## 2. Materials and Methods

### 2.1. Experimental Materials

The mulberry varieties used in this experiment were Ewu No. 1 (EW1) and Husan No. 32 (H32), both of which were established with a central trunk training system in 1996. The planting density was 133 cm × 67 cm. The experiment was conducted at the Mulberry Germplasm Resource Garden in Hubei Province, where test plots with level ground and uniform soil fertility were selected. The experiment was repeated three times, and within each plot, three mulberry trees with similar trunk circumference, crown diameter, and overall growth vigor were selected as the test materials. The mulberry trees were pruned in summer, and fertilization and irrigation were managed according to high-yield mulberry orchard cultivation practices. Additionally, pest and disease control measures were strengthened, and a thinning of buds was performed to ensure a well-structured leaf canopy. The soil in the experimental field was typical yellow-brown soil with moderate fertility, slightly acidic properties with a pH ranging from 5.6 to 6.5, and an organic matter content that was above average.

### 2.2. Measurement of Photosynthetic Physiological Parameters

The net photosynthetic rate (Pn), stomatal conductance (Gs), intercellular CO_2_ concentration (Ci), and transpiration rate (Tr) of mulberry leaves were assessed using the LI-6400XT portable photosynthesis system (LI-COR, Lincoln, NE, USA). These measurements were conducted in mid-May, with data collected every 2 h between 6:00 a.m. and 6:00 p.m. on three well-illuminated, healthy leaves located at the top of each sample tree. Additionally, the Pn-PAR and Pn-Ci response curves were analyzed, with photosynthetically active radiation (PAR) levels ranging from 0 to 1800 μmol/(m^2^·s) and CO_2_ concentrations set across 12 gradients (ranging from 0 to 1500 μmol/mol).

### 2.3. Measurement of Chlorophyll Fluorescence Parameters

Chlorophyll fluorescence parameters were measured using the LI-6400 portable photosynthesis system (LI-COR, Lincoln, NE, USA), which was equipped with a fluorescence chamber. Leaf selection for these measurements was consistent with those used in photosynthetic rate assessments. The measurements were conducted on 15–16 May (the average light intensity on 15 May and 16 May was 1549.605 lux and 1538.767 lux, respectively), one day after the diurnal photosynthesis evaluations. Prior to the experiment, the selected mulberry leaves were wrapped in aluminum foil for 22–24 h to ensure complete dark adaptation. The average temperature and humidity on 15 May and 16 May were 32.11 °C with 68.466% humidity and 32.94 °C with 66.982% humidity, respectively.

On the morning of the experiment (5:30–7:00 a.m.), the minimum initial fluorescence (Fo) was measured under detection light, ensuring that all PSII reaction centers were fully open. A strong saturating pulse was then applied to determine the maximum fluorescence (Fm) in the dark-adapted leaves. The leaves were subsequently exposed to continuous actinic light (PPFD = 1200 μmol·m^−2^·s^−1^) for 30 min to achieve steady-state fluorescence (Fs). Following this, an additional saturating pulse (PPFD = 2000 μmol·m^−2^·s^−1^) was applied to measure the maximum fluorescence after light adaptation (Fm′). After six measurements, the actinic and detection lights were turned off, and far-red light was applied to obtain the initial fluorescence after light adaptation (Fo′). The instrument automatically calculated additional chlorophyll fluorescence parameters, including the maximum quantum efficiency of PSII (Fv/Fm), the actual quantum efficiency of PSII (ΦPSII), the photochemical quenching coefficient (qP), the non-photochemical quenching (NPQ), and the electron transport rate (ETR) of PSII. These parameters were calibrated using the following formulas:

- Maximum quantum efficiency of PSII, Fv/Fm = (Fm − Fo)/Fm
- Actual quantum efficiency of PSII, ΦPSII = ΔF/Fm′ = (Fm′ − Fs)/Fm′
- Apparent electron transport rate, ETR = 0.5 × 0.84 × ΦPSII × PPFD
- Photochemical quenching coefficient, qP = (Fm′ − Fs)/(Fm′ − Fo′)
- Non-photochemical quenching, NPQ = (Fm − Fm′)/Fm′

### 2.4. Measurement of Physicochemical Parameters

The sample was ground into a fine powder using liquid nitrogen, and approximately 0.5 g of the powdered sample was weighed. To this, 5 mL of distilled water and approximately 250 mg of calcium carbonate powder were added. The mixture was thoroughly ground in the dark or under low light conditions, then transferred to a 50 mL glass tube. The mortar was rinsed with 95% ethanol, and the rinsate was combined with the contents of the glass tube, bringing the total volume to 50 mL using the extraction solution. The sample was allowed to extract in darkness (or wrapped in foil) for 3 h, ensuring that the residue turned completely white. If decolorization was incomplete, the extraction process continued until full decolorization was achieved. The absorbance was then measured at 663 nm and 645 nm, recorded as A663 and A645, respectively. The chlorophyll content was calculated using the following formulas:

- Chlorophyll a content (mg/g) = 0.02 × (12.7 × A663 − 2.69 × A645) × N/M
- Chlorophyll b content (mg/g) = 0.02 × (22.9 × A645 − 4.68 × A663) × N/M
- Total chlorophyll content (mg/g) = 0.02 × (20.21 × A645 + 8.02 × A663) × N/M

where the following apply:

- A663 and A645 are the absorbance values at 663 nm and 645 nm, respectively.
- N is the dilution factor.
- M is the fresh weight of the sample.

One gram of the sample was accurately weighed and placed in a centrifuge tube, and an equal volume of pre-cooled 0.1 mol/L PBS solution was added. The mixture was thoroughly mixed and then centrifuged at 2500 r/min. The supernatant was reserved and adjusted to a final volume of 10 mL for subsequent assays. For the enzyme assays of RuBP carboxylase (RuBisCo), Rubisco activase (RCA), phosphoenolpyruvate carboxylase (PEPC), NADP malic enzyme (NADP-ME), and carbonic anhydrase (CA), the necessary strips were prepared from aluminum foil bags, which had been equilibrated to room temperature for 20 min. The unused strips were sealed and stored at 4 °C. Wells were set up with standards and samples by adding 50 μL of different concentration standards to the standard wells and 10 μL of the test sample plus 40 μL of dilution buffer to the sample wells. No reagents were added to the blank wells. Subsequently, 100 μL of HRP-labeled detection antibody was added to each well, except the blank wells. The plate was sealed and incubated at 37 °C for 60 min in a water bath or incubator. After incubation, the liquid was discarded, the wells were blotted dry with absorbent paper, and each well was filled with wash buffer. The wells were allowed to sit for 1 min before discarding the wash, and this washing process was repeated five times. Next, 50 μL of substrates A and B was added to each well, and the plate was incubated at 37 °C in the dark for 15 min. After incubation, 50 μL of stop solution was added to each well, and the optical density (OD) values were measured at 450 nm within 15 min.

### 2.5. Total RNA Extraction and Transcriptomic Sequencing

Total RNA was extracted from EW1-10, EW1-12, EW1-16, H32-10, H32-12, and H32-16 using TRIzol Reagent (TaKaRa Bio Inc., Dalian, China) according to the manufacturer’s protocol. The quantity and quality of the extracted RNA were assessed using an Agilent Bioanalyzer 2100 (Agilent Technologies, Santa Clara, CA, USA) and a Nanodrop ND-2000 spectrophotometer (Thermo Fisher Scientific, Waltham, MA, USA). Further RNA quantification was performed using a Qubit 2.0 fluorometer (Life Technologies, Carlsbad, CA, USA). Equal amounts of RNA from individual samples were pooled, reverse-transcribed into cDNA, and subsequently sequenced as paired-end 125 bp reads using the Illumina HiSeq 2300 platform (Illumina, San Diego, CA, USA) by BGI Genomics (Shenzhen, China). RNA sequencing was performed in triplicate for each sample.

### 2.6. De Novo Assembly and Annotation

Following RNA sequencing, the quality of the raw reads was assessed and visualized using FastQC version 0.11.5. Low-quality reads and those containing adaptors were filtered out using the NGS QC Toolkit [25]. The de novo transcriptome was assembled using the Trinity platform, which integrates the Inchworm, Chrysalis, and Butterfly modules, with a k-mer size of 25 [26]. To obtain nonredundant unigenes, sequence splicing and redundancy were further minimized using the TGI Clustering Tool version 2.1 [27]. The unigenes were annotated using BLASTx version 2.2.26, with an E-value cutoff of 10^−5^. Additional annotation of the unigenes was performed against several databases, including the NCBI nonredundant protein sequences (NR), NCBI nucleotide sequences (NT), Swiss-Prot, Pfam, Kyoto Encyclopedia of Genes and Genomes (KEGG), euKaryotic Orthology Groups (KOG), and Gene Ontology (GO).

### 2.7. Differential Expressed Gene (DEGs) and Pathway Enrichment Analysis

The Bowtie2 software was employed to align the reads to the reference genes [28]. The read counts for each sample were subsequently analyzed using the DESeq2 package in R to identify differentially expressed genes (DEGs) [29]. Gene expression levels were quantified using FPKM (fragments per kilobase of transcript per million mapped reads) values, with differential expression defined by a q-value < 0.05 and |log2(fold change)| > 1. GO enrichment analysis of the DEGs was performed using the GOseq package, which adjusts for gene length bias [30]. Significantly enriched pathways were identified using KOBAS 2.0, with a false discovery rate (FDR) ≤ 0.05 [31].

## 3. Results

### 3.1. Photosynthetic Physiological Assessment

**Figure 1:**
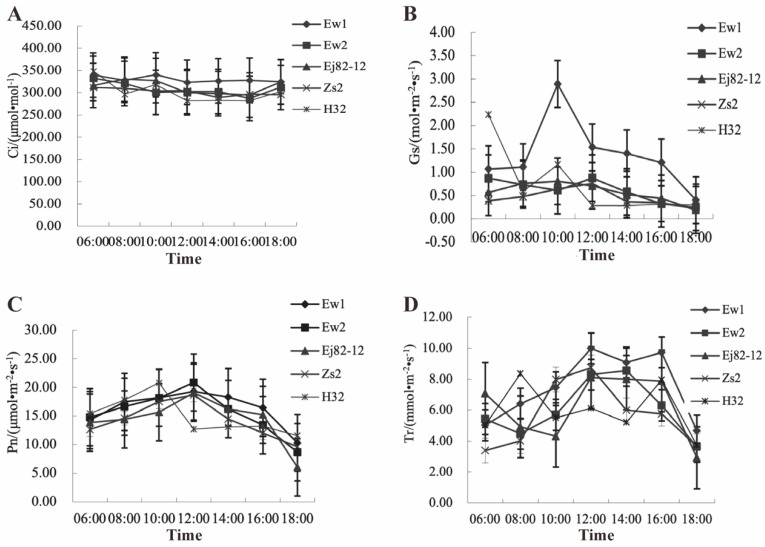
The evaluation of photosynthetic physiological parameters in the five mulberry species over time. (**A**) The intercellular CO_2_ concentration (Ci). (**B**) The stomatal conductance (Gs). (**C**) The photosynthetic rate (Pn). (**D**) The transpiration rate (Tr). Indication: Ew1, Ewu No. 1; Ew2, Ewu No. 2; Zs2, Zhushan No. 2; Ej82-12, Ejian 82-12; H32, Husan No. 32.

The analysis of intercellular CO_2_ concentration (Ci), stomatal conductance (Gs), photosynthetic rate (Pn), and transpiration rate (Tr) across five mulberry species reveals distinct physiological strategies in response to diurnal environmental changes. For intercellular CO_2_ concentration, Ew1 (ranging from 339.39 to 324.29 μmol/mol) and Ew2 (ranging from 331.91 to 311.77 μmol/mol) likely maintain higher Ci values throughout the day, which supports their higher photosynthetic rates and indicates efficient CO_2_ assimilation. In contrast, Zs2 (ranging from 311.49 to 295.21 μmol/mol) and Ej82-12 (ranging from 323.49 to 294.37 μmol/mol) show a more pronounced decline in Ci during midday, likely due to reduced CO_2_ availability caused by stomatal closure and decreased photosynthetic demand. H32 (ranging from 348.26 to 281.31 μmol/mol) exhibits a significant drop in Ci, particularly around midday, suggesting that this species is less capable of maintaining its CO_2_ levels, leading to reduced photosynthesis (Figure 1A).

Regarding stomatal conductance (Gs) (Figure 1B), Ew1 and Ew2 display higher and more stable Gs values, indicating a more efficient gas exchange system, allowing these species to sustain higher photosynthetic rates. This balance between water loss and CO_2_ uptake suggests a robust adaptation to environmental fluctuations. Zs2 and Ej82-12 show a more significant decline in Gs around midday, suggesting that these species respond to environmental stress by closing their stomata more, limiting CO_2_ uptake and reducing photosynthesis. H32 shows a sharp decline in Gs, especially during midday, indicating a strong stomatal response to avoid excessive water loss but at the cost of reduced photosynthetic activity.

In terms of the photosynthetic rate (Pn) (Figure 1C), Ew1 (average 16.31 μmol/m^2^/s) and Ew2 (average 15.51 μmol/m^2^/s) likely exhibit increased Pn throughout the day, especially in the morning and afternoon. This suggests that these species are more efficient in capturing light and converting it into chemical energy, possibly due to their better adaptation to local environmental conditions. Zs2 (average 14.20 μmol/m^2^/s) and Ej82-12 (average 14.32 μmol/m^2^/s) might exhibit a more pronounced midday depression in Pn, reflecting a higher sensitivity to environmental stressors such as intense light and high temperatures, indicating a lower efficiency in maintaining photosynthesis under midday conditions. H32, with the lowest average Pn values (12.97 μmol/m^2^/s), is particularly vulnerable to photoinhibition and environmental stress, resulting in reduced photosynthetic efficiency. Its ΦPSII value (0.127) is notably lower than that of Ew1 (0.189), indicating fewer electrons are being transferred through PSII per photon absorbed. This lower ΦPSII suggests damage to the photosynthetic apparatus caused by excessive light exposure.

For the transpiration rate (Tr), Ew1 (average 9.53 mmol/m^2^/s) and Ew2 (average 8.41 mmol/m^2^/s) exhibit higher Tr values, particularly in the midday, suggesting higher water-use efficiency, which supports their higher photosynthetic rates (Figure 1D). Zs2 (average 7.36 mmol/m^2^/s) and Ej82-12 (average 8.05 mmol/m^2^/s) show a drop in Tr during midday, reflecting a more conservative water-use strategy, helping these species avoid excessive water loss but also limiting their photosynthetic potential. H32 shows the lowest Tr values (average 5.65 mmol/m^2^/s), especially during midday, indicating a highly conservative water-use strategy, which may be helpful for survival under extreme conditions but limits photosynthetic efficiency.

### 3.2. The Pn-PAR and Pn-Ci Curve Analyses

**Figure 2:**
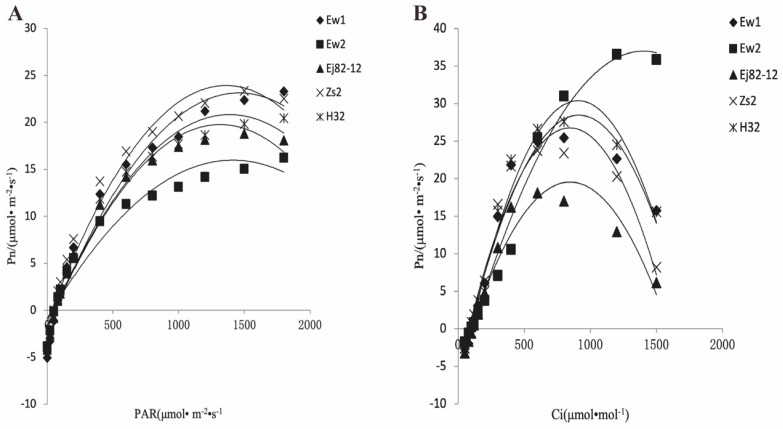
Pn-PAR and Pn-Ci curve analyses in the five mulberry species over time. (**A**) The Pn-PAR curve. (**B**) The Pn-Ci curve. Indication: Pn, photosynthetic rate; PAR, photosynthetically active radiation; Ci, intercellular CO_2_ concentration; Ew1, Ewu No. 1; Ew2, Ewu No. 2; Zs2, Zhushan No. 2; Ej82-12, Ejian 82-12; H32, Husan No. 32.

The Pn-PAR curve generally exhibits an initial increase in the photosynthetic rate (Pn) as photosynthetically active radiation (PAR) intensifies, reflecting the light-dependent reactions of photosynthesis. As light intensity rises, the rate of photosynthesis escalates until it reaches a saturation point, beyond which further increases in PAR do not significantly enhance Pn (Figure 2A). Ew1 and Ew2 likely demonstrate higher photosynthetic rates at lower PAR levels, achieving saturation more rapidly than the other species. This observation suggests that Ew1 and Ew2 are more efficient at capturing and utilizing light energy, potentially indicative of a more robust light-harvesting complex or greater overall efficiency within their photosynthetic apparatus. Conversely, Zs2 and Ej82-12 may reach saturation at higher PAR levels, implying a requirement for greater light intensity to attain maximum photosynthetic efficiency. This trait could indicate that these species are adapted to environments with higher light availability, though they may be less efficient under low-light conditions. H32, which exhibits a lower photosynthetic rate across all PAR levels and a delayed saturation point, appears less efficient at converting light energy into chemical energy. This inefficiency might suggest limitations within the light-harvesting complex or a slower response to increasing light intensity.

The Pn-Ci curve typically shows an increase in the photosynthetic rate with rising Ci, reflecting the availability of CO_2_ for carboxylation within the Calvin cycle. As Ci increases, photosynthesis continues to rise until it reaches a plateau, where other factors, such as the capacity of photosynthetic enzymes, become limiting. Ew1 and Ew2 likely maintain higher photosynthetic rates across a broad range of Ci values, indicating efficient CO_2_ assimilation (Figure 2B). This efficiency suggests that Ew1 and Ew2 possess a robust carboxylation mechanism, possibly due to higher Rubisco activity. In contrast, Zs2 and Ej82-12 may exhibit a more gradual increase in photosynthesis with rising Ci, suggesting a slower or less efficient carboxylation process. This could imply that these species are less efficient at utilizing available CO_2_, potentially limiting their growth under fluctuating CO_2_ conditions. H32, with the lowest photosynthetic rate across Ci levels, likely experiences limitations in CO_2_ uptake or utilization, possibly due to lower Rubisco activity or other biochemical constraints within the Calvin cycle.

### 3.3. Chlorophyll Fluorescence Parameter Assessment

**Figure 3:**
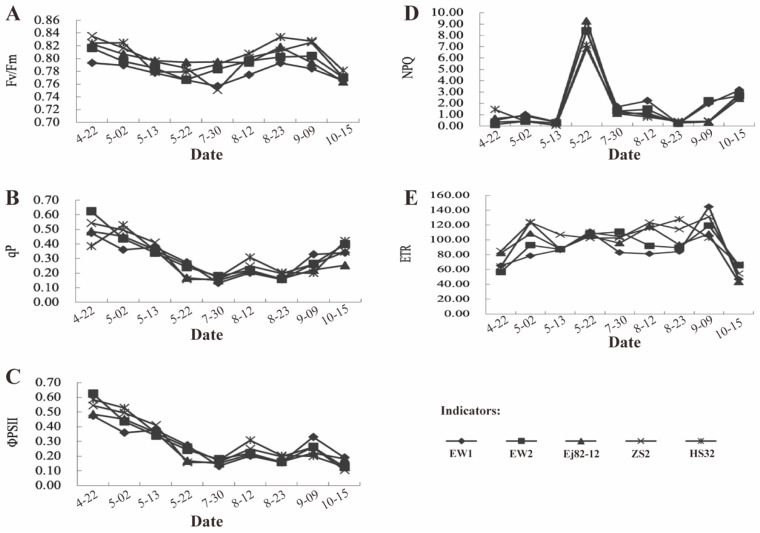
The chlorophyll fluorescence parameter assessment in the five mulberry species over time. (**A**) The maximum quantum efficiency of PSII (Fv/Fmin). (**B**) The photochemical quenching coefficient (qP). (**C**) The actual quantum efficiency of PSII (ΦPSII). (**D**) The non-photochemical quenching (NPQ). (**E**) The apparent electron transport rate (ETR). Indication: Pn, photosynthetic rate; PAR, photosynthetically active radiation; Ci, intercellular CO_2_ concentration; Ew1, Ewu No. 1; Ew2, Ewu No. 2; Zs2, Zhushan No. 2; Ej82-12, Ejian 82-12; H32, Husan No. 32.

The Fv/Fm ratio reflects the maximum efficiency of PSII photochemistry under dark-adapted conditions and serves as a reliable indicator of a plant’s potential photosynthetic performance. The Fv/Fm value, which measures the maximum efficiency of photosystem II (PSII) under dark-adapted conditions, is a key indicator of a plant’s photosynthetic health. Values near 0.83 indicate healthy PSII function with minimal stress, while values below 0.5 suggest significant stress, likely caused by factors such as drought, extreme temperatures, or nutrient deficiencies. Ew1 and Ew2 likely maintain high Fv/Fm values across all measurement dates, suggesting minimal photoinhibition and a healthy, efficient PSII system (Figure 3A). In contrast, Zs2 and Ej82-12 may exhibit slight declines in Fv/Fm on certain dates, indicating mild photoinhibition or stress that compromises PSII efficiency. H32, with its lower Fv/Fm values, particularly on specific dates, appears more susceptible to photoinhibition, which could reduce its overall photosynthetic efficiency.

The qP parameter reflects the proportion of PSII reaction centers that are open and actively engaged in photochemistry. High qP values in Ew1 and Ew2 indicate that a significant proportion (*p* < 0.05) of their PSII reaction centers participate in photochemical processes, suggesting efficient light utilization. For Zs2 and Ej82-12, slightly lower qP values may suggest a smaller proportion of open reaction centers, potentially due to partial closure as a protective response. In H32, even lower qP values could indicate that many reaction centers are closed or non-functional, possibly due to stress-induced damage or inefficiency (Figure 3B).

The ΦPSII parameter indicates the efficiency of PSII under light-adapted conditions, reflecting actual photochemical performance. The higher ΦPSII values observed in Ew1 and Ew2 suggest that these species maintain efficient photochemistry under light conditions, which correlates with their potentially higher photosynthetic rates (Figure 3C). Zs2 and Ej82-12 may exhibit moderate ΦPSII values, indicating effective but less efficient photochemistry compared to Ew1 and Ew2. In contrast, lower ΦPSII values in H32 may suggest reduced efficiency in utilizing absorbed light for photochemistry, potentially due to increased energy dissipation or stress.

The non-photochemical quenching (NPQ) measures the dissipation of excess energy as heat, a protective mechanism against light-induced stress. The lower NPQ values in Ew1 (ranging from 0.367 to 8.391) and Ew2 (ranging from 0.138 to 8.366) suggest that these species efficiently use most of their absorbed light for photochemistry, with minimal energy dissipated as heat. Zs2 (ranging from 0.044 to 6.850) and Ej82-12 (ranging from 0.263 to 9.277), with moderate NPQ values, may balance between photochemical use and protective heat dissipation, indicating moderate stress or a protective response. In H32 (ranging from 0.069 to 7.151), slightly higher NPQ values could indicate greater energy dissipation as heat, a protective response to avoid photodamage but also an indication of less efficient light energy use for photosynthesis (Figure 3D).

The electron transport rate (ETR) measures the rate at which electrons are transported through the photosynthetic electron transport chain, directly linked to overall photosynthetic activity (Figure 3E). Higher ETR values in Ew1 and Ew2 indicate robust electron transport, supporting the high photosynthetic activity in these species. The higher ETR in Ew1 and Ew2 correlates with their increased photosynthetic rates, suggesting a direct relationship. Zs2 and Ej82-12, with moderate ETR values, suggest effective but not maximal electron transport rates, correlating with moderate photosynthetic efficiency. The lower ETR values in H32 may suggest limitations in electron transport, potentially due to stress or less efficient photosynthetic machinery.

### 3.4. Physicochemical Parameter Assessment

**Figure 4:**
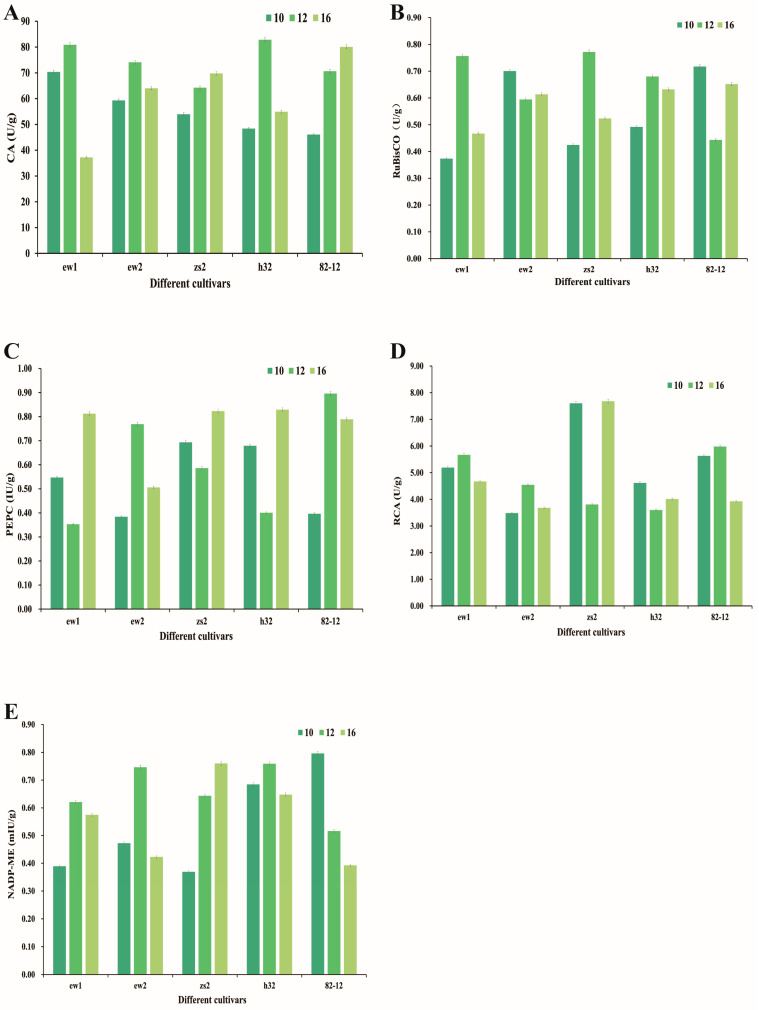
The physicochemical parameter assessment in the five mulberry species over time. (**A**) Carbonic anhydrase (CA). (**B**) RuBP carboxylase (RuBisCo). (**C**) Phosphoenolpyruvate carboxylase (PEPC). (**D**) Rubisco activase (RCA). (**E**) NADP malic enzyme (NADP-ME). Error bars represent the standard error of the mean (SEM). Indication: Ew1, Ewu No. 1; Ew2, Ewu No. 2; Zs2, Zhushan No. 2; Ej82-12, Ejian 82-12; H32, Husan No. 32.

The activity of carbonic anhydrase (CA) shows variability across different mulberry cultivars and time points. Overall, CA activity tends to increase in the afternoon, reaching higher levels at 4 p.m. compared to 10 a.m. and 12 p.m., particularly in the cultivars ew2 and Ej82-12 (Figure 4A). This suggests a potential diurnal regulation of CA activity, likely influenced by light intensity and photosynthetic demand. RuBisCo activity also exhibits fluctuations across the time points, with a general trend in lower activity at midday (12 p.m.) and higher activity at 4 p.m., particularly in the cultivars Zs2 and H32 (Figure 4B). This relatively lower midday activity could be associated with midday depression, a phenomenon where photosynthetic activity decreases due to factors like high temperature and photoinhibition. PEPC activity appears to follow a trend similar to CA, with higher enzyme activity observed at 4 p.m. across most cultivars, particularly ew1, zs2, and H32 (Figure 4C). This increase may be related to the enzyme’s role in the C4 and CAM photosynthesis pathways, where it facilitates carbon fixation during periods of enhanced photosynthetic capacity.

RCA activity shows notable differences across cultivars, with pronounced increases at 4 p.m. in Ew2 and Zs2, while other cultivars exhibit relatively stable activity levels (Figure 4D). RCA is essential for maintaining RuBisCo’s catalytic activity by facilitating the removal of inhibitory sugar phosphates, and its increased activity at 4 p.m. may contribute to the observed recovery of RuBisCo activity. The activity of NADP-ME shows higher levels at 4 p.m. for most of the cultivars, with a distinct increase in H32 and Ej82-12 (Figure 4E). NADP-ME is involved in malate decarboxylation in the C4 and CAM pathways, releasing CO_2_ for the Calvin cycle. Its increased activity in the late afternoon suggests an adaptation to optimize carbon utilization during periods when photosynthetic efficiency can be maximized, potentially compensating for earlier reductions in RuBisCo activity.

**Figure 5:**
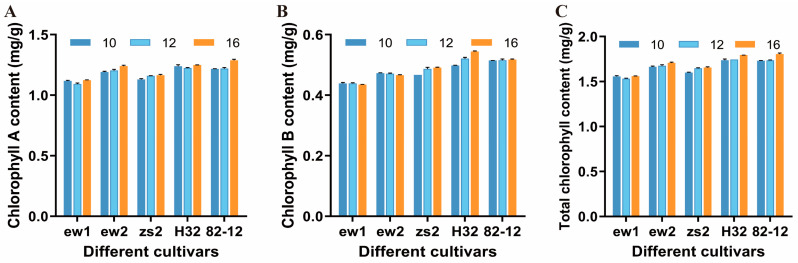
The chlorophyll parameter assessment in the five mulberry species over time. (**A**) Chlorophyll A content. (**B**) Chlorophyll B content. (**C**) Total chlorophyll content. Error bars represent the standard error of the mean (SEM). Indication: Ew1, Ewu No. 1; Ew2, Ewu No. 2; Zs2, Zhushan No. 2; Ej82-12, Ejian 82-12; H32, Husan No. 32.

The chlorophyll A content across the five different mulberry cultivars (Ew1, Ew2, Zs2, H32, and Ej82-12) shows some variation over the time points measured (10 a.m., 12 p.m., and 4 p.m.). For most cultivars, chlorophyll A content remains relatively stable across the time points, with a slight increase observed at 4 p.m. (Figure 5A). This pattern suggests that chlorophyll A levels may be more influenced by environmental factors later in the day, possibly due to changes in light intensity or temperature, which could enhance chlorophyll synthesis or stability. The chlorophyll B content demonstrates a more consistent trend across all cultivars, with slight fluctuations but no significant changes between 10 a.m., 12 p.m., and 4 p.m. (Figure 5B). The data suggest that chlorophyll B may be less sensitive to diurnal variations compared to chlorophyll A, or that its synthesis and degradation may be regulated more tightly throughout the day. The total chlorophyll B content mirrors the trends observed in chlorophyll A, with an overall increase observed at 4 p.m. for all cultivars, particularly in H32 (Figure 5C). This increase suggests that the combined effects of chlorophyll A and B contribute to a higher total chlorophyll level later in the day, which may be an adaptive response to optimize photosynthetic capacity as light conditions change.

### 3.5. RNA-seq Bioinformatic Analysis

Eighteen RNA libraries were generated from the Ew1 and H32 mulberry species at different time points, specifically 10:00 a.m., 12:00 p.m., and 4:00 p.m. The RNA sequencing yielded between 40 and 56 million reads per library, with consistently high-quality scores (Q30, 88–92%) and GC contents ranging from 45% to 46%. Table 1 provides comprehensive statistics summarizing the RNA sequencing data for each library at the different time points. The RNA-seq data obtained from Ew1 and H32 across these time points demonstrate high quality, characterized by consistent sequencing depth, quality scores, and GC content. The high alignment ratios indicate that the majority of the reads were successfully mapped to the reference genome, ensuring a robust foundation for comprehensive transcriptomic analysis. These results offer a solid basis for the further exploration of gene expression differences between species and time points, which could yield valuable insights into diurnal gene regulation and species-specific adaptations.

### 3.6. Functional Annotation and Classification of the Transcriptome Data

**Figure 6:**
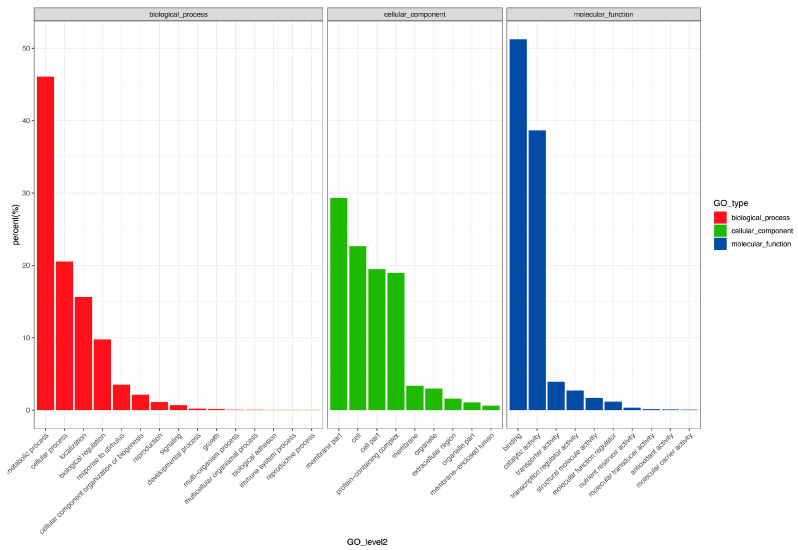
The functional classification of unigenes in Gene Ontology (GO) from the Ew1 and H32 mulberry species at different time points (10:00, 12:00, and 16:00).

A total of 21,759 unigenes were identified from the transcriptome data and subsequently annotated against six major functional databases: GO, KEGG, KOG, Nr, Pfam, and SwissProt. The annotations yielded 10,630 unigenes (48.85%) in GO, 5904 (27.13%) in KEGG, 11,767 (54.07%) in KOG, 21,699 (99.72%) in Nr, 17,461 (80.24%) in Pfam, and 16,536 (75.99%) in SwissProt. The annotated unigenes were classified into three main functional categories in the GO classification: biological process, molecular function, and cellular component. In the biological process category, metabolic processes (including carbohydrate metabolic process, lipid metabolic process, nitrogen compound metabolic process, tryptophan metabolic process, and trehalose metabolic process) were prominently represented (46.08%), while membrane part terms dominated the cellular component category. The most represented terms in the molecular function category were binding terms (51.22%) (Figure 6).

**Figure 7:**
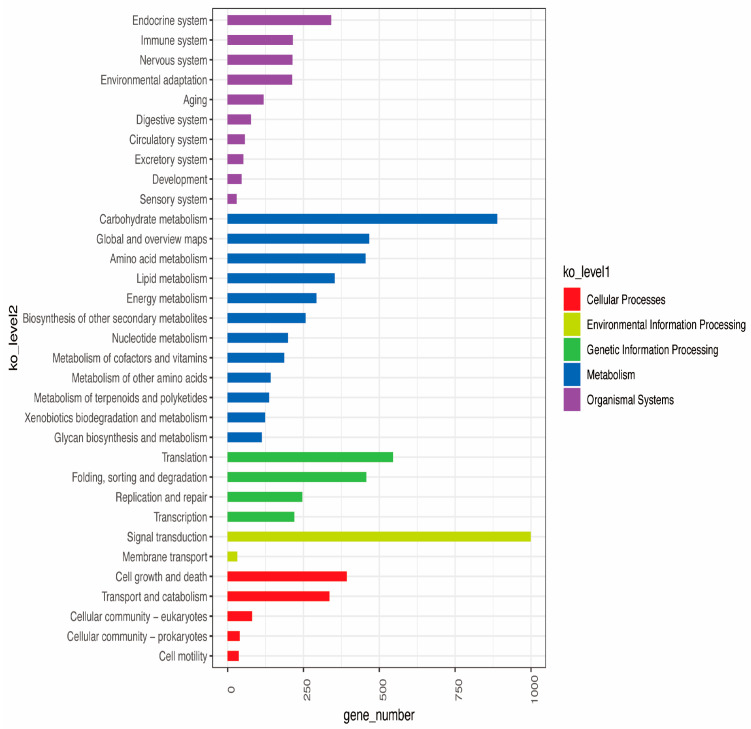
The functional classification of unigenes in Kyoto Encyclopedia of Genes and Genomes (KEGG) from the Ew1 and H32 mulberry species at different time points (10:00, 12:00, and 16:00).

In the KEGG pathway analysis, the annotated unigenes were mapped to five major pathways: organismal systems, metabolism, genetic information processing, environmental information processing, and cellular processes. Specifically, 342 unigenes were associated with the endocrine system, which regulates hormonal balance and signaling, essential for coordinating stress responses, growth, and development in mulberry. Additionally, 889 unigenes were linked to carbohydrate metabolism, supporting energy production and storage to meet the plant’s demands during photosynthesis and stress adaptation, particularly under midday depression. Translation, represented by 545 unigenes, underscores the importance of protein synthesis in maintaining cellular functions and enabling adaptive responses to environmental stressors. Signal transduction, involving 999 unigenes, facilitates cell-to-environment communication, allowing the plant to initiate appropriate physiological and molecular responses to stress. Finally, 393 unigenes were tied to cell growth and death signaling pathways, which balance cell proliferation and programmed cell death, critical for tissue repair, growth regulation, and survival under adverse conditions (Figure 7). Together, these pathways illuminate the molecular mechanisms underlying mulberry’s adaptability and resilience, offering insights to improve productivity and stress tolerance. The transcriptome data provide a comprehensive overview of gene expression in the studied mulberry species, highlighting significant roles in metabolic activity, signal transduction, and cellular processes. The extensive annotation across multiple databases strengthens the functional assignments and indicates that the identified unigenes are critical in plant growth, development, and environmental responses. Further exploration of these pathways, particularly those involved in metabolism and signal transduction, could yield deeper insights into the molecular mechanisms governing these processes in mulberry species. This information could be pivotal for breeding programs aimed at enhancing stress tolerance, growth efficiency, and other desirable traits.

### 3.7. Differential Gene Expression (DEG) Analysis

**Figure 8:**
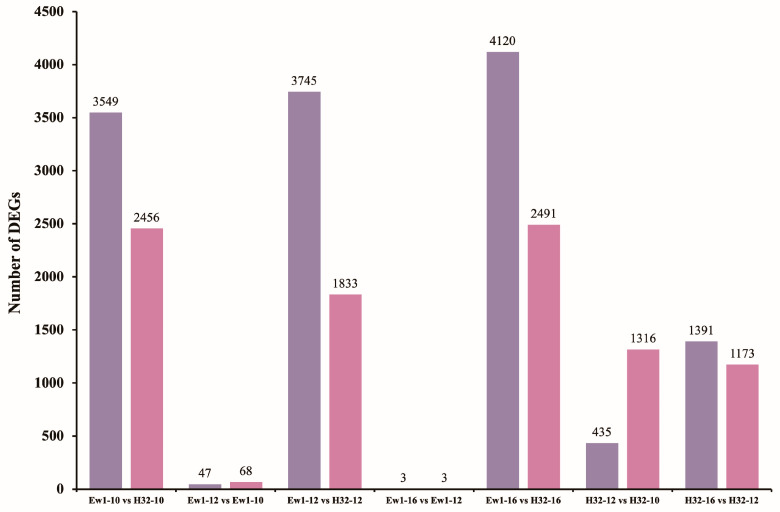
The number of up- and downregulated DEGs in the Ew1-10 vs. H32-10, Ew1-12 vs. Ew1-10, Ew1-12 vs. H32-12, Ew1-16 vs. Ew1-12, Ew1-16 vs. H32-16, H32-12 vs. H32-10, and H32-16 vs. H32-12 groups.

Gene expression analysis identified 22,630 differentially expressed genes (DEGs) across seven comparative groups between the Ew1 and H32 mulberry species at different time points: 10:00 a.m., 12:00 p.m., and 4:00 p.m. The number of upregulated DEGs in each comparison was as follows: 3549 for Ew1-10 vs. H32-10, 47 for Ew1-12 vs. Ew1-10, 3745 for Ew1-12 vs. H32-12, 3 for Ew1-16 vs. Ew1-12, 4120 for Ew1-16 vs. H32-16, 435 for H32-12 vs. H32-10, and 1391 for H32-16 vs. H32-12. Conversely, the number of downregulated DEGs in each comparison was 2456, 68, 1833, 3, 2491, 1316, and 1173, respectively, for the same groups. Ew1 demonstrated stable gene expression over time, with minimal changes in DEGs within itself (e.g., 47 upregulated DEGs for Ew1-12 vs. Ew1-10 and only 3 for Ew1-16 vs. Ew1-12), suggesting a consistent and well-regulated response to environmental stress. In contrast, H32 showed greater variability, with more significant changes in DEGs (e.g., 435 upregulated DEGs for H32-12 vs. H32-10 and 1391 for H32-16 vs. H32-12), indicating a more dynamic but potentially less efficient stress response. Comparatively, Ew1 exhibited a higher number of upregulated DEGs than H32 in species-to-species comparisons, such as Ew1-10 vs. H32-10 (3549 upregulated DEGs) and Ew1-16 vs. H32-16 (4120 upregulated DEGs). This suggests that Ew1 employs a robust and proactive strategy by activating stress-responsive pathways. Conversely, H32 had fewer upregulated DEGs and more downregulated ones, reflecting a conservative approach focused on reducing metabolic activity and prioritizing survival under stress. These findings indicate that Ew1’s gene expression is tailored for resilience and efficient adaptation to stress, while H32 relies on a reactive, resource-conserving strategy. These differences highlight distinct molecular strategies for coping with environmental challenges. Figure 8 provides a detailed summary of the DEG counts, including both up- and downregulated genes, for the Ew1 and H32 mulberry species at the specified time points.

### 3.8. Signaling Pathways Involved in Mulberry Varieties Regarding Midday Depression

**Figure 9:**
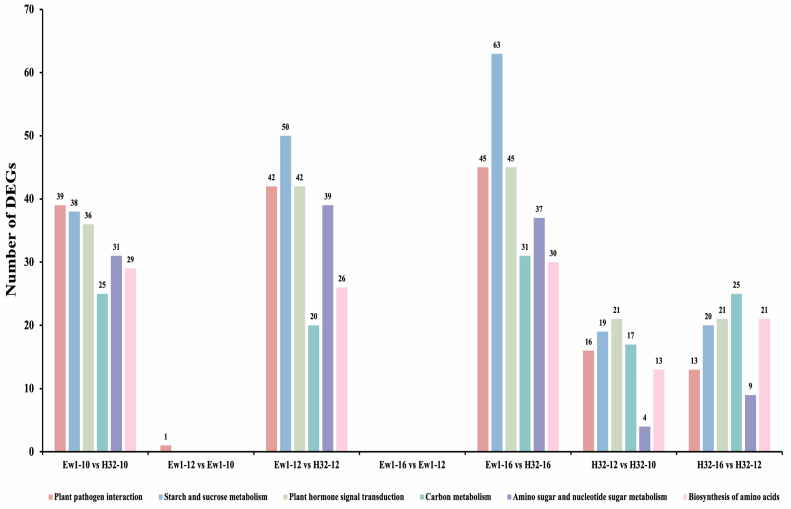
The signaling pathways and the number of DEGs involved in the Ew1 and H32 mulberry species at different time points (10:00 a.m., 12:00 p.m., and 4:00 p.m.) associated with midday depression.

A comparative KEGG enrichment analysis was performed for each comparative group between the Ew1 and H32 mulberry species at different time points: 10:00 a.m., 12:00 p.m., and 4:00 p.m. The KEGG enrichment analysis identified several key signaling pathways associated with midday depression in mulberry species, including plant–pathogen interactions, starch and sucrose metabolism, plant hormone signal transduction, carbon metabolism, amino sugar and nucleotide sugar metabolism, and biosynthesis of amino acids. These pathways were enriched in most of the comparative groups between Ew1 and H32 at different time points (10:00 a.m., 12:00 p.m., and 4:00 p.m.), indicating their importance in the physiological responses related to midday depression. The starch and sucrose metabolism pathway exhibited the highest number of DEGs across the species and time points, with 19 to 63 DEGs involved. This indicates that carbohydrate metabolism, particularly the management of energy reserves like starch and sucrose, plays a significant role in determining how mulberry species cope with midday stress. The biosynthesis of amino acids pathway had fewer DEGs (13 to 30), suggesting that while amino acid biosynthesis is important, it may be less central to the immediate responses associated with midday depression compared to carbohydrate metabolism. Understanding the identified signaling pathways provides actionable insights for breeding programs aimed at enhancing mulberry resilience and productivity. Pathways like starch and sucrose metabolism, plant hormone signaling, and carbon metabolism are pivotal for managing energy balance and stress responses during midday depression. The enrichment of DEGs in starch and sucrose metabolism highlights the potential for optimizing carbohydrate use to sustain photosynthesis under stress. By leveraging these pathways, breeders can develop mulberry varieties with improved energy efficiency, stress tolerance, and growth regulation. These insights offer a practical framework for breeding strategies to ensure high productivity and sustainable agricultural practices. Figure 9 illustrates the signaling pathways and the number of DEGs involved in the Ew1 and H32 mulberry species at different time points associated with midday depression.

### 3.9. Genes Involved in Mulberry Varieties Regarding Midday Depression

Based on the gene expression analysis of mulberry species Ew1 and H32, the identified genes—jacalin-related lectin 3, o-acyltransferase WSD1, CASP-like protein 1E2, ABC transporter G family member 11, TIR-NBS-LRR-like protein, and hydroquinone glucosyltransferase—appear to be involved in the physiological mechanisms underlying midday depression in mulberry species. The upregulation of genes involved in water conservation, energy management, and structural integrity (e.g., jacalin-related lectin 3, O-acyltransferase WSD1, and CASP-like protein 1E2) highlights the plant’s effort to protect itself from midday stress. These genes likely contribute to the plant’s ability to conserve water, maintain photosynthetic efficiency, and ensure energy balance during periods of high environmental stress, such as midday. Conversely, the downregulation of genes involved in transport, pathogen defense, and detoxification (e.g., ABC transporter G family member 11, TIR-NBS-LRR, and hydroquinone glucosyltransferase) suggests a strategic reallocation of resources, with the plant deprioritizing certain pathways in favor of more immediate survival strategies. Table 2 summarizes the expression level (fold change) of the annotated DEGs involved in midday depression across different mulberry species and time points. It is important to note that while our RNA-Seq analysis provides valuable insights into the molecular mechanisms under investigation, we were unable to perform qPCR validation due to limited sample availability. This limitation should be taken into consideration when interpreting the results, as RNA-Seq data, although highly informative, may occasionally include inherent variability. We recommend the cautious interpretation and understanding of the RNA-Seq findings, and we acknowledge the need for additional validation in future studies to further confirm the observed trends and enhance the reliability of these conclusions.

## 4. Discussion

The five mulberry species studied exhibit distinct physiological strategies to cope with diurnal changes in environmental conditions. Ew1 and Ew2 appear more robust, maintaining higher photosynthetic rates, stomatal conductance, and transpiration rates throughout the day. This suggests better adaptation to fluctuating conditions, likely due to more efficient water and CO_2_ management. In contrast, Zs2 and Ej82-12 seem more sensitive to environmental stress, showing greater midday depression in photosynthesis and lower stomatal conductance and transpiration rates, reflecting a water-conserving strategy at the expense of reduced photosynthetic activity. H32, the most conservative species in terms of stomatal conductance and transpiration, is the least productive under stressful midday conditions, prioritizing water conservation over photosynthetic efficiency, which may be advantageous in extreme environments but limits overall productivity. These differences highlight the diverse strategies employed by mulberry species to balance photosynthesis with water conservation, reflecting their varying degrees of adaptation to environmental conditions.

The analysis of midday depression in mulberry species reveals distinct physiological responses to diurnal environmental changes. Ew1 and Ew2 maintain a higher Ci throughout the day, supporting efficient CO_2_ assimilation and increased Pn. Their stable Gs reflects robust gas exchange, enabling them to balance CO_2_ uptake and water loss, which results in a higher Tr [10]. These traits suggest superior adaptation to fluctuating light and temperature, enabling these species to mitigate midday depression more effectively. In contrast, Zs2 and Ej82-12 show a more pronounced midday decline in Ci and Gs, likely due to increased stomatal closure to conserve water under midday stress [13]. This strategy reduces CO_2_ availability, limiting photosynthesis and transpiration. These species exhibit a greater sensitivity to environmental stressors, such as intense light and high temperatures, leading to more substantial midday depression in photosynthesis [32]. While this water-conserving strategy aids in survival, it comes at the cost of lower photosynthetic activity during midday. H32 is the most conservative species, displaying the lowest Pn, Gs, and Tr values, especially at midday. Its sharp decline in Ci and stomatal conductance suggests that H32 prioritizes water conservation over photosynthetic efficiency, making it well suited for extreme environments but less productive overall [33]. The midday depression in H32 reflects a strong protective response to environmental stress, particularly photoinhibition and water loss, limiting its capacity to sustain photosynthesis. These results highlight the diverse strategies mulberry species employ to cope with diurnal changes, with Ew1 and Ew2 exhibiting greater resilience, while Zs2, Ej82-12, and H32 adopt more conservative, water-conserving approaches.

The relationships observed in the Pn-PAR and Pn-Ci curves provide valuable insights into the photosynthetic efficiency and adaptability of the different mulberry species. Ew1 and Ew2 emerge as the most efficient species, exhibiting higher photosynthetic rates both at lower light intensities and across varying CO_2_ concentrations, suggesting superior adaptability to a range of environmental conditions, potentially enhancing their resilience and productivity. Although Zs2 and Ej82-12 are effective, they may require more optimal conditions—such as higher light or CO_2_ levels—to achieve their maximum photosynthetic potential. H32, by contrast, appears the least efficient across both parameters, indicating it may be more sensitive to environmental fluctuations and less capable of sustaining high photosynthetic rates under suboptimal conditions. These differences underscore the varying strategies and adaptations among the mulberry species, which are crucial considerations for selecting species for cultivation in diverse environmental settings or for breeding programs aimed at enhancing photosynthetic efficiency.

The physiological analysis of midday depression in the Ew1 and H32 mulberry species at different time points (10:00 a.m., 12:00 p.m., and 4:00 p.m.) highlights significant variations in their photosynthetic efficiency and adaptive strategies. The Pn-PAR and Pn-Ci curves reveal that Ew1, along with its close relative Ew2, exhibits superior photosynthetic efficiency. These species achieve photosynthetic saturation at lower PAR levels and maintain higher Pn across a broad range of Ci values. This resilience is likely due to their robust light-harvesting complexes and efficient CO_2_ assimilation mechanisms, potentially supported by higher Rubisco activity. As a result, Ew1 and Ew2 demonstrate the ability to mitigate the effects of midday stress, such as high light intensity and elevated temperatures, allowing them to sustain higher photosynthetic rates during midday, whereas many other species experience significant declines [34].

Conversely, H32 exhibits lower photosynthetic efficiency, with a delayed saturation point and reduced Pn and Ci values. This indicates inefficiencies in both light capture and CO_2_ utilization, likely due to limitations in the Calvin cycle or reduced Rubisco activity. As a result, H32 is more vulnerable to midday depression, struggling to sustain photosynthesis under environmental stress. Its conservative strategy, with a lower Gs and Tr, reflects a greater focus on water conservation rather than maintaining photosynthetic output, making it less productive overall. Comparing these results to other species, C3 crops such as wheat and rice often show a similar midday depression due to stomatal closure and photoinhibition [12,13]. However, C4 plants like maize are more efficient in mitigating these effects, maintaining higher Pn under midday stress due to their specialized photosynthetic pathways. Ew1 and Ew2 align more closely with the resilience seen in C4 plants, while H32 mirrors the vulnerability of stress-sensitive C3 species. In summary, the superior adaptability and resilience of Ew1 and Ew2 make them ideal candidates for cultivation in a variety of environmental conditions. H32, however, would benefit from targeted breeding efforts aimed at improving its photosynthetic efficiency and stress tolerance.

The analysis of the photochemical parameters in Ew1 and H32 mulberry species at different time points (10:00 a.m., 12:00 p.m., and 4:00 p.m.) reveals distinct physiological responses to midday depression, a phenomenon where photosynthetic activity declines due to environmental stress like high light intensity and heat. Ew1 and Ew2 maintain high Fv/Fm ratios throughout the day, suggesting minimal photoinhibition and the efficient protection of PSII from damage [35]. This resilience is reflected in their ability to sustain high photosynthetic rates even during midday stress, as indicated by their high qP and ΦPSII values. These parameters reveal that a large proportion of their PSII reaction centers remain functional and actively engaged in photochemistry, supporting efficient CO_2_ assimilation and their higher photosynthetic output.

In contrast, H32 exhibits significantly lower Fv/Fm values, indicating a greater susceptibility to photoinhibition, especially at midday when light intensity and temperature are at their peak. The low qP and ΦPSII values in H32 suggest that many PSII reaction centers are inactive or damaged, leading to a sharp decline in photosynthetic efficiency. This vulnerability to stress, combined with high NPQ values, indicates that H32 relies heavily on energy dissipation as heat to protect against further photodamage [36]. However, this strategy comes at the cost of reduced electron transport, as reflected in the lower ETR, and consequently lower overall photosynthetic performance. Comparatively, C4 species like maize are known for their higher resistance to photoinhibition and ability to maintain efficient photosynthesis under stress, owing to their specialized carbon fixation pathways [37]. Ew1 and Ew2 exhibit a similar resilience, suggesting that their robust PSII activity and efficient use of light energy make them better adapted to fluctuating environmental conditions. H32, on the other hand, aligns more with sensitive C3 crops like wheat, which often suffer from midday depression due to stomatal closure and photoinhibition. In conclusion, the superior adaptability of Ew1 and Ew2 makes them ideal candidates for cultivation in environments prone to midday stress, while the vulnerability of H32 suggests the need for targeted breeding efforts to improve its photosynthetic efficiency and stress tolerance.

The observed increase in CA activity in the late afternoon (4 p.m.) across the mulberry cultivars, particularly ew2 and 82-12, suggests a diurnal regulation mechanism. CA is involved in catalyzing the interconversion of CO_2_ and bicarbonate, thus playing a crucial role in supplying CO_2_ for photosynthesis [38]. The elevated CA activity later in the day indicates that mulberry may enhance CO_2_ hydration during periods of peak light availability to support photosynthesis as light intensity declines. Similar trends have been reported in other plant species such as Arabidopsis thaliana, where CA activity peaks in the afternoon, likely compensating for earlier reductions in stomatal conductance and providing a sustained CO_2_ supply during recovery from midday depression [39,40]. The reduction in RuBisCo activity at midday and its subsequent increase at 4 p.m. is indicative of a typical midday depression response, where RuBisCo efficiency is reduced due to heat-induced deactivation and increased photorespiration [41,42]. RCA, which facilitates the activation of RuBisCo by removing inhibitory sugar phosphates, shows increased activity in the late afternoon, especially in cultivars ew2 and zs2. This pattern suggests that RCA may be a key factor in overcoming midday inhibition and reactivating RuBisCo [43].

The increase in PEPC activity at 4 p.m., particularly in cultivars ew1, zs2, and H32, may be related to its role in C4 and CAM photosynthesis. While mulberry is not a C4 species, PEPC still plays a role in refixing respiratory CO_2_. Its elevated activity during the late afternoon suggests that PEPC may be compensating for reduced RuBisCo activity and helping sustain carbon fixation during recovery from midday depression [44]. This adaptive response is similar to that observed in Zea mays, a C4 species, where PEPC activity peaks later in the day, optimizing carbon fixation under conditions of fluctuating light and temperature [45,46]. The NADP-ME increased activity at 4 p.m., particularly in H32 and 82-12, supports the idea that NADP-ME may facilitate the release of CO_2_ for the Calvin cycle during periods of declining light. The role of NADP-ME in mulberry under diurnal changes may reflect a strategy to optimize the balance between C3 photosynthesis and photorespiration. This trend is consistent with the patterns observed in CAM species like maize, where NADP-ME activity increases towards the evening, enhancing CO_2_ availability for photosynthesis as stomatal conductance gradually recovers [47].

The relatively stable chlorophyll A content throughout the day, with a slight increase at 4 p.m., suggests that mulberry cultivars may maintain chlorophyll levels to optimize light absorption during periods of changing light conditions. This stability may be an adaptive response to prevent photodamage during midday depression while enhancing photosynthetic capacity later in the day [48,49]. The more stable levels of chlorophyll B across all time points indicate that light-harvesting complexes may maintain their efficiency throughout the day, contributing to sustained photosynthetic performance despite diurnal stress. The overall increase in total chlorophyll content at 4 p.m. across all cultivars, especially H32, supports the notion that mulberry enhances its light-harvesting potential during the late afternoon. This response may prepare the photosynthetic machinery for the anticipated decline in light availability, thus maximizing energy capture [50,51]. The observed trends in enzyme activities and chlorophyll content in mulberry cultivars share similarities with other C3 species that experience midday depression. In these species, midday reductions in photosynthetic enzyme activities are followed by a late-afternoon recovery phase. However, mulberry exhibits certain unique adaptations, such as relatively stable chlorophyll B content and pronounced increases in CA and PEPC activities in the afternoon, which may provide additional resilience to environmental stress.

The consistent pattern of more upregulated DEGs in Ew1 compared to H32 across all time points suggests that Ew1 may be more resilient or responsive to environmental changes throughout the day. The results also indicate that Ew1 maintains a relatively stable gene expression profile, with minimal variation between time points. In contrast, H32 exhibits greater variation, particularly a notable downregulation of genes at midday, which could be a strategy to cope with midday stress but might also limit its overall productivity compared to Ew1. The observed differences in DEGs between the two species and across different time points likely reflect underlying genetic and physiological variations that influence how these species adapt to daily environmental fluctuations. The gene expression analysis reveals significant differences in how the Ew1 and H32 mulberry species manage their physiological processes throughout the day. Ew1 appears to be more stable and active, while H32 shows more pronounced changes, particularly downregulation around midday. These findings provide valuable insights into the temporal and species-specific dynamics of gene expression, which could inform breeding strategies and guide the selection of mulberry species for different environmental conditions.

The gene expression analysis reveals significant insights into how Ew1 and H32 mulberry species respond to midday depression and diurnal environmental changes. Ew1 demonstrates a greater number of upregulated differentially expressed genes (DEGs) across all time points, particularly during midday, indicating a robust physiological response to environmental stress such as high light intensity and heat [52]. The substantial gene activity observed in comparisons like Ew1-12 vs. H32-12 and Ew1-16 vs. H32-16 highlights Ew1’s ability to maintain photosynthesis and productivity despite harsh midday conditions. This heightened resilience likely involves the upregulation of stress-responsive genes related to photosynthesis, heat-shock proteins, and antioxidant defense [53], which enables Ew1 to effectively manage midday stress and sustain growth.

In contrast, H32 exhibits a pronounced downregulation of DEGs, particularly at midday (12:00 PM), signaling a reduction in metabolic and photosynthetic activity. This downregulation suggests that H32 adopts a more conservative approach, likely involving stomatal closure or reduced metabolic processes to avoid water loss and photodamage. The minimal fluctuation in gene expression observed in Ew1 between the time points (e.g., Ew1-12 vs. Ew1-10 and Ew1-16 vs. Ew1-12) further underscores its stable adaptation to environmental fluctuations, suggesting that Ew1 possesses more efficient regulatory mechanisms, such as enhanced photosynthetic gene regulation and stress tolerance pathways. This stability contrasts sharply with H32, whose more dynamic but less efficient gene expression pattern resembles stress-sensitive C3 plants like wheat, which often exhibit midday depression [13]. On the other hand, C4 plants like maize maintain higher gene activity under stress [37], similar to the resilience seen in Ew1. Hence, Ew1 demonstrates superior adaptability and stable gene expression, making it more resilient to environmental fluctuations, while H32 relies on defensive mechanisms that compromise midday productivity, suggesting the need for targeted breeding strategies to enhance its stress tolerance.

The KEGG enrichment analysis highlights key molecular pathways involved in managing midday depression in Ew1 and H32 mulberry species, offering insights into their physiological responses to environmental stress at different time points (10:00 a.m., 12:00 p.m., and 4:00 p.m.). The consistent enrichment of pathways related to starch and sucrose metabolism, plant hormone signal transduction, and carbon metabolism across time points underscores their central role in coping with midday stress. Starch and sucrose metabolism, showing the highest number of DEGs [54], points to the importance of carbohydrate management for energy storage and mobilization under stress. This suggests that these species utilize carbohydrate reserves to sustain metabolic functions and mitigate the effects of intense light and heat during midday.

Ew1 maintains a stable physiological state throughout the day, with minimal fluctuations in gene expression between time points (e.g., Ew1-12 vs. Ew1-10 and Ew1-16 vs. Ew1-12). This stability likely reflects more efficient regulatory mechanisms that support consistent carbohydrate metabolism and hormone signaling. Such stability allows Ew1 to minimize the effects of midday depression, which aligns with the resilience typically observed in C4 plants like maize [37]. By contrast, H32 exhibits greater variability in gene expression, especially during midday, which suggests less efficient metabolic regulation. The limited involvement of DEGs in amino acid biosynthesis in both species indicates that while this pathway supports stress responses, it is secondary to carbohydrate metabolism in its role in the immediate responses to midday depression.

When compared with other plant species, the metabolic stability observed in Ew1 mirrors the efficient CO_2_ utilization seen in C4 plants, which are more adept at handling midday stress [55]. C3 species like wheat, on the other hand, often experience significant midday depression due to limitations in carbon metabolism and stomatal regulation [56]. H32’s fluctuating metabolic activity is more characteristic of such stress-sensitive C3 plants. Overall, Ew1’s superior resilience to midday stress, driven by stable energy and hormone pathway regulation, contrasts with H32’s vulnerability, underscoring the potential for targeted breeding to enhance midday stress tolerance in mulberry and other crops.

The gene expression analysis of Ew1 and H32 mulberry species highlights contrasting molecular strategies in response to midday depression, which is triggered by environmental stressors such as high light intensity and elevated temperatures. Ew1 demonstrates the upregulation of genes like jacalin-related lectin 3, O-acyltransferase WSD1, and CASP-like protein 1E2, which play critical roles in water retention, structural integrity, and energy management [57,58,59]. These gene functions suggest that Ew1 is equipped with mechanisms to optimize water use and maintain photosynthetic activity even under midday stress. This effective resource allocation allows Ew1 to sustain productivity and minimize midday depression by stabilizing its physiological processes throughout the day.

In contrast, H32 shows a downregulation of genes associated with transport, pathogen defense, and detoxification, such as ABC transporter G family member 11, TIR-NBS-LRR, and hydroquinone glucosyltransferase. This reflects a more defensive strategy in H32, where it conserves water and energy by reducing non-essential activities. However, this trade-off results in diminished metabolic and photosynthetic efficiency, particularly at midday when environmental stress peaks [60]. H32’s approach, while effective for short-term survival, limits its overall productivity compared to Ew1. The stability of the gene expression in Ew1 across different time points (10:00 a.m., 12:00 p.m., and 4:00 p.m.) suggests that Ew1 has evolved robust regulatory mechanisms that maintain physiological resilience during midday stress. This stability contrasts with the greater variability in H32, which requires more significant shifts in gene expression to manage stress, leading to a less consistent photosynthetic performance. Comparing these results to other species, C4 plants like maize show a similar resilience, maintaining stable gene expression and efficient photosynthesis during midday stress [61]. Ew1 resembles the stability seen in C4 species, while H32 mirrors the midday sensitivity typical of C3 species. This analysis underscores the potential of breeding programs aimed at enhancing midday stress tolerance in mulberry species by focusing on traits similar to those seen in Ew1. Lastly, we emphasize that while our RNA-Seq analysis offers valuable insights into the molecular mechanisms under investigation, the absence of qPCR validation due to limited sample availability is a notable limitation. This should be considered when interpreting the results, as RNA-Seq data, though highly informative, may exhibit inherent variability. We advise a cautious approach to understanding these findings and recognize the importance of additional validation in future studies to confirm the observed trends and strengthen the reliability of our conclusions.

## 5. Conclusions

This study underscores the critical differences in how Ew1 and H32 mulberry species respond to midday depression, with Ew1 demonstrating superior resilience through higher photosynthetic rates and stable physiological parameters. The upregulation of genes associated with stress adaptation in Ew1 suggests robust mechanisms for mitigating adverse midday conditions. Conversely, H32 exhibited a more pronounced midday depression, marked by lower photosynthetic activity and gene downregulation, reflecting a conservative approach that limits productivity. The molecular insights provided can inform breeding strategies aimed at enhancing stress tolerance and photosynthetic efficiency in mulberry, potentially improving crop performance in environments prone to midday stress.

## Figures and Tables

**Table 1:** Summary statistics for the RNA-sequencing data from each sample.

Sample Name	Reads Number	Q30 (%)	GC Content (%)	Total Alignment Ratio (%)
Ew1-10-1	44,244,072	88	46	88.36
Ew1-10-2	46,497,380	88	45	86.48
Ew1-10-3	457,58,114	88	45	88.63
Ew1-12-1	46,571,626	88	45	90.24
Ew1-12-2	43,834,562	89	45	89.99
Ew1-12-3	52,297,044	89	46	90.73
Ew1-16-1	46,256,696	89	46	90.06
Ew1-16-2	49,410,778	89	46	89.03
Ew1-16-3	51,546,568	89	46	89.72
H32-10-1	56,238,320	89	46	92.71
H32-10-2	55,119,798	89	46	92.09
H32-10-3	51,069,830	89	46	93.07
H32-12-1	45,635,198	89	46	92.66
H32-12-2	46,995,546	89	46	92.52
H32-12-3	40,395,218	89	46	92.14
H32-16-1	47,639,000	92	45	91.92
H32-16-2	46,990,202	91	46	90.75
H32-16-3	40,586,496	91	46	92.40

**Table 2:** The expression level (fold change) of the annotated DEGs involved in midday depression across different mulberry species and time points.

Genes	Ew1-10 vs. H32-10	Ew1-12 vs. Ew1-10	Ew1-12 vs. H32-12	Ew1-16 vs. Ew1-12	Ew1-16 vs. H32-16	H32-12 vs. H32-10	H32-16 vs. H32-12
*Jacalin-related lectin 3*	+17.579	0	+16.207	0	+18.569	0	+1.1720
*O-acyltransferase WSD1*	+11.427	0	+11.483	0	+11.624	−2.697	+1.186
*CASP-like protein 1e2*	+9.896	0	+8.824	0	+9.396	0	0
*ABC transporter G family member 11*	−10.245	0	−12.031	0	−8.459	−1.636	−3.621
*TIR-NBS-LRR-like protein*	−9.967	0	−10.867	0	−10.919	0	0
*Hydroquinone glucosyltransferase*	−9.411	0	−9.530	0	−8.933	−1.709	−1.163

## Data Availability

The original contributions presented in this study are included in the article, further inquiries can be directed to the corresponding author.
